# Supplementary material for: Obesity impairs cardiolipin-dependent mitophagy and therapeutic intercellular mitochondrial transfer ability of mesenchymal stem cells
Source: Cell Death Dis. 2023 May 13;14(5):324. doi: 10.1038/s41419-023-05810-3 (PMC10181927; doi:10.1038/s41419-023-05810-3)
Supplement: Supplementary file 1 — Supplementary Information [file 41419_2023_5810_MOESM1_ESM.docx]

**Supplementary Figure Legends**

**Figure S1: Characterization of obesity features in HFD mice model: (A)**Body weight of the mice fed regular diet (lean mice) or high-fat diets (obese mice) was measured after 16 weeks. The weight is presented in grams/mice*.*(**B**) Fasting glucose was measured in the blood of lean and obese mice. (**C, D**) Similarly, total cholesterol and triglyceride content was measured in the serum. Data is presented as Mean±SEM. *****P* < 0.001; ***P* < 0.01.

**Figure S2: MSC-Ob show cell death under culture conditions with a trend toward an increase in cellular senescence**

**(A)**Flow cytometry analysis of stem cell markers expressed by MSC-L and MSC-Ob. The Y axis represents the cell count and the x-axis represents the type of fluorescent antibody used detected by the corresponding laser. For Sca-1 and CD44 PE conjugated antibody was used and for CD11b FOTC conjugated antibody was used. (**B**) Representative images show cells stained with propidium iodide (PI) and calcein green. The number of PI-positive cells counted per 100 cells in the images *(B)*. The PI-positive cells indicate cell death (*Right panel*). (**C**) Scatter plot showing the percentage of death measured by staining the cells with PI and annexin V (AV). (**D**) Cell proliferation was measured by staining the cells with CFSE. (**E**) Representative phase-contrast images of cells stained with Senescence-associated β-galactosidase staining (SA-β-gal) are shown in blue. Red arrow heads show the β-gal positive cells. The number of SA-β-gal positive cells were counted and represented as percent senescent cells per 1000 cells counted. Data is shown as Mean±SEM. ****P* < 0.005; ns (non-significant). Scale bars: B: 50 µm; E: 200 µm.

**Figure S3**

**Miro1 expression and TNT formation do not change between MSC-L and MSC-Ob:**

**(A)**Miro1 RT-qPCR was performed in RNA extracted from the cells and plotted as a relative expression by normalizing with β-actin expression. **(B)**Representative images show tunneling nanotube formation between MSCs and MLE12. MSCs were transduced with mito-GFP (green) and co-cultured with CTDR stained MLE12 for 24 hrs before fixing and staining with Phalloidin (red). The Blue arrowhead shows the TNT formation between MSC and MLE12**. (C)** TNT quantitation between MSCs and MLE12. Data is shown as Mean±SEM. *****P* < 0.001; ***P* < 0.001; ns (non-significant). Scale bars: 10 µm.

**Figure S4**

**MSC-Ob show decline in mitochondrial bioenergetics:**

**(A)**Basal respiration was measured by monitoring the oxygen consumption rate (OCR) in MSC-L and MSC-Ob, respectively. (**B-D**) Similarly, maximal respiration, spare respiratory capacity, ATP production was measured. Data is shown as Mean±SEM. ***P* < 0.001. **P* < 0.05.

**Figure S5**

**LC3-dependent autophagosome formation is reduced in MSC-Ob.**

(**A**) Immunoblot of mitochondrial and cytosolic extracts showing α-tubulin expression in cytosolic extract and COX-IV expression in the mitochondrial extract (**B**) Representative images of the cells stained for LC3 (red), Tom20 (green), and DAPI (blue) treated with DMSO (Veh) or FCCP 10µM) for 1 hr. (*Below panel*) Line scan shows colocalization in the regions shown highlighted in images. (*right panel*) show degree of colocalization between LC3 and Tom20 represented as Mander’s coefficient. (**C**) Similarly, cells were treated with 10µM antimycin A (AMA) for 0, 15, and 120 m. The part of this image is also shown in main *Figure 2F*. (**D**) Representative images of cells transduced with LC3-YFP vector (green) and stained for Tom20 (red) and DAPI (blue). The cells were treated with DMSO (Veh) or FCCP (10µM). Mander’s coefficient was calculated to determine the degree of colocalization between LC3-YFP and Tom20 (*right panel*). Data is shown as Mean±SEM. *****P* < 0.001; ****P* < 0.005; **P* < 0.05. Scale bars: 10 µm.

**Figure S6**

**The expression of core autophagy markers is not altered in the MSC-Ob:**

**(A)**Immunoblots of Atg5, Atg7, and β-actin in the cell lysates prepared from MSCs. (**B**) Similarly, immunoblotting was done for total and phosphorylated forms of Beclin along with β-actin loading as control. (**C**) Densitometry analysis of the blots shown in *A, B*. (**D**) Autophagy pathway analysis was done using the RT2 profiler PCR array. The upregulated genes are shown in yellow dots, and black dots represent genes that did not significantly change expression. Data is shown as Mean±SEM. ns (non-significant).

**Figure S7**

**Starvation induces autophagosome formation and autophagy of p62:**

**(A)** Representative images of p62 colocalization with LC3 in cells cultured under normal conditions and starvation (SRV). (**B**) Similarly, cells were stained with lysotracker deep red (LTDR). The degree of colocalization was calculated between P62 with LC3 or LTDR, respectively (below panels). (**C**) The autophagosome formation was calculated in cells stained for LC3 under normal and SRV conditions. Data is shown as Mean±SEM. ****P* < 0.005; ***P* < 0.01; **P* < 0.05; ns (non-significant). Scale bars: 10 µm.

**Figure S8**

**Reduced cardiolipin colocalization with LC3:**

**(A).** Cardiolipin species detected by LC-MS in MSC-L, MSC-Ob and MSC-Ob^PQQ^ groups. The cardiolipin species which are significantly different between MSC-L vs MSC-Ob and MSC-Ob vs MSC-Ob^PQQ^ are shown in *Figure 3K* and *Figure S11A.* The cardiolipin species which are significantly different are shown in *Figure 3K*. **(B).** Histogram showing the colocalisation of LC3 (red) with NAO (green), representing the autophagosomes and cardiolipins respectively. **(C).** Representative images of cells were treated with Veh or FCCP (10 µM) for 2 hrs. To induce depolarization, MSC-L and MSC-Ob were treated with Veh or FCCP and stained for NAO (green) and LC3 (red). The insets below show the region of interest (ROI). Scale bars: 10 µm and 5 µm (ROI).

**Figure S9**

**Human MSCs show mitochondrial dysfunction and reduced cardiolipin content upon FFA treatment:**

**(A)**hMSCs isolated from the bone marrow of normal healthy individuals were analyzed for the expression of stem cell markers. Flow cytometry was performed, and the data is represented as % expression with respect to the isotype control.**(B)**mtROS was measured in hMSC treated with Veh (DMSO) or free fatty acids (FFA) for 24 hrs. The analysis was done by flow cytometry using mitoSOX red. (**C**) Representative images of hMSCs stained with MTR (red) and Hoechst (blue) to find the changes in mitochondrial morphology. (**D**) Mitochondrial mass was calculated in the cells (*C*) stained with MTR. (**E**) Cardiolipin content was measured in hMSCs by flow cytometry and represented as the bar graph. (**F**) Representative images of cells stained with NAO (green) and LC3 (red). The cells were treated with Veh or FCCP (µM) for 1 hr before imaging. (*Right panel*) shows corresponding Mander’s coefficient of the images shown in *F*. Data is shown as Mean±SEM. *****P* < 0.001; ***P* < 0.01; **P* < 0.05. Scale bars: 10 µm.

** Figure S10**

**PQQ treatments restore mitochondrial function and ultrastructural changes:**

(**A**) Representative histograms of cells showing mtROS. MSC-Ob were treated with PQQ (30µM) for the indicated days with media change after every 2 days and supplemented with fresh PQQ (shown as T: no. of treatments). (**B**) Representative images show changes in mitochondrial shape and size. (**C**) Representative EM images showing mitochondrial ultrastructural changes with disrupted cristae indicated by red arrow head and healthy mitochondria by blue arrow head. The number of cristae were quantified and presented as percentage of imperfect cristae per 100 cristae counted (*right panel*). Data is shown as Mean±SEM. ***P* < 0.01; **P* < 0.05. Scale bars: C: 10 µm; D: 0.1µm.

**Figure S11**

**PQQ restores cardiolipin and LC3 colocalization:**

(**A**) LC-MS data showing bar graphs of all the detected cardiolipin species in MSC-L, MSC-Ob and MSC-Ob^PQQ^ groups. The same panel shown in *Figure 4K* for the cardiolipin species which are significantly different between various groups. **(B)** Bar graph showing the signal quantitation of NAO (represents cardiolipin content) in MSCs treated with Veh or PQQ. (**C**) Representative images showing colocalization of cardiolipin stained with NAO (green) with LC3 (red) in cells treated with Veh or FCCP (10 µM for 1 hr.). The insets below show the respective ROIs of the square dotted area. (**D**) The corresponding Mander’s coefficient of the images shows the extent of colocalization. The histogram panel *C* is same as shown in the *Figure 3L* which is without PQQ data. (**E**) Bar graph showing the signal quantitation of NAO (represents cardiolipin content) in hMSCs treated with Veh, FFA and FFA+PQQ (**F**) Representative images of hMSCs treated with PQQ and stained with NAO (green) and LC3 (red) with insets below the respective ROIs. (**G**) Corresponding Mander’s coefficient representation, the extent of colocalization between cardiolipin and LC3. Data is shown as Mean±SEM. *****P* < 0.001; ****P* < 0.005; ***P* < 0.01; **P* < 0.05. Scale bars: C: 10 µm; Insets: 5µm.

**Figure S12**

**MSC-Ob treated with PQQ restore airway mechanics, airway remodeling, and airway epithelial cell damage:** (**A**) AHR in Ova-induced allergic airway inflammation model transplanted with MSCs obtained from lean or HFD mice and treated with various concentrations and time points of PQQ. The groups 2mg and 4mg refer to the HFD mice which were fed PQQ for 15 days before harvesting MSCs. (**B**) H&E images showing the airway infiltration of cells (blue arrowheads). (**C**) Inflammatory score representing the extent of airway inflammatory cell infiltration. (**D**) Representative images of tissue sections stained with PAS and pseudocolored. The pink color represents the mucus secretion, while the blue color is the representation of nuclei. The images were subjected to quantitative analysis and represented as integrated density *(right panel)*. (**E**) Representative images showing TUNEL staining in bronchial epithelial cells. Brown represents the TUNEL positive cells (cell death) per 100 nuclei counted stained with hematoxylin (blue). Mean±SEM. *****P* < 0.001; ****P* < 0.005; ***P* < 0.01; **P* < 0.05; ns (non-significant). Scale bars: B: 200 µm; D: 100 µm; E: 50 µm.

**References:**

1. D. Ryu *et al.*, Urolithin A induces mitophagy and prolongs lifespan in C. elegans and increases muscle function in rodents. *Nat. Med.* **22**, 879-888 (2016).

2. Y. Choi *et al.*, Enhancement of Mesenchymal Stem Cell-Driven Bone Regeneration by Resveratrol-Mediated SOX2 Regulation. *Aging Dis.* **10**, 818-833 (2019).

3. Y. Zhang *et al.*, Adult mesenchymal stem cell ageing interplays with depressed mitochondrial Ndufs6. *Cell Death Dis.* **11**, 1075 (2020).

4. W. Chowanadisai *et al.*, Pyrroloquinoline quinone stimulates mitochondrial biogenesis through cAMP response element-binding protein phosphorylation and increased PGC-1alpha expression. *J. Biol. Chem.* **285**, 142-152 (2010).

5. E. F. Fang *et al.*, NAD(+) augmentation restores mitophagy and limits accelerated aging in Werner syndrome. *Nat Commun* **10**, 5284 (2019).

6. Y. T. Wu *et al.*, Dual role of 3-methyladenine in modulation of autophagy via different temporal patterns of inhibition on class I and III phosphoinositide 3-kinase. *J. Biol. Chem.* **285**, 10850-10861 (2010).

7. C. J. Li, L. Y. Sun, C. Y. Pang, Synergistic protection of N-acetylcysteine and ascorbic acid 2-phosphate on human mesenchymal stem cells against mitoptosis, necroptosis and apoptosis. *Sci. Rep.* **5**, 9819 (2015).
