# Supplementary material for: Obesity impairs cardiolipin-dependent mitophagy and therapeutic intercellular mitochondrial transfer ability of mesenchymal stem cells
Source: Cell Death Dis. 2023 May 13;14(5):324. doi: 10.1038/s41419-023-05810-3 (PMC10181927; doi:10.1038/s41419-023-05810-3)
Supplement: Supplementary file 2 — Supplemental File_Uncropped WB [file 41419_2023_5810_MOESM2_ESM.pptx]

## Slide 1
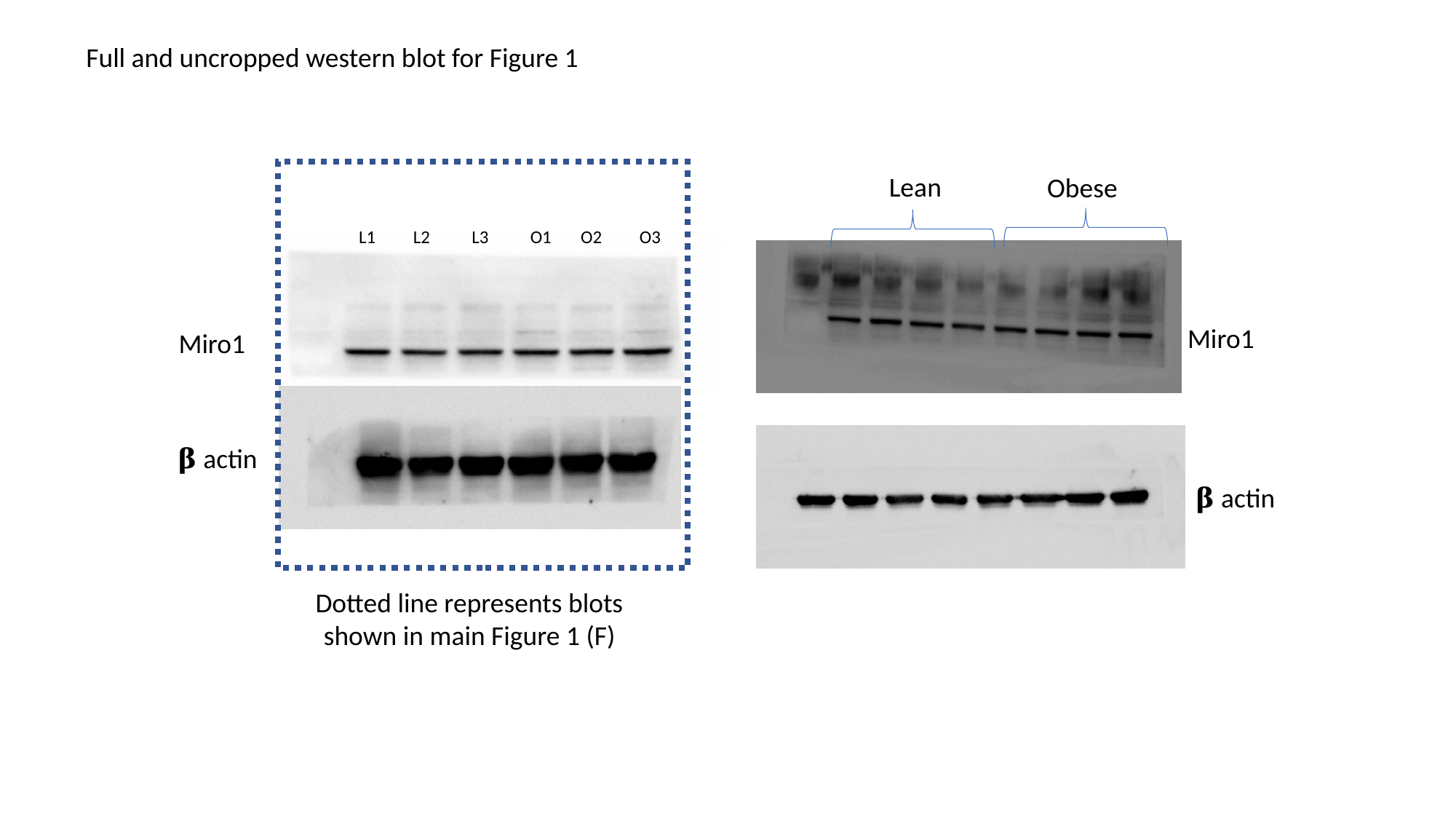

Full and uncropped western blot for Figure 1
Lean
Obese
 L1 L2 L3 O1 O2 O3
Miro1
Miro1
𝛃 actin
𝛃 actin
Dotted line represents blots shown in main Figure 1 (F)

## Slide 2
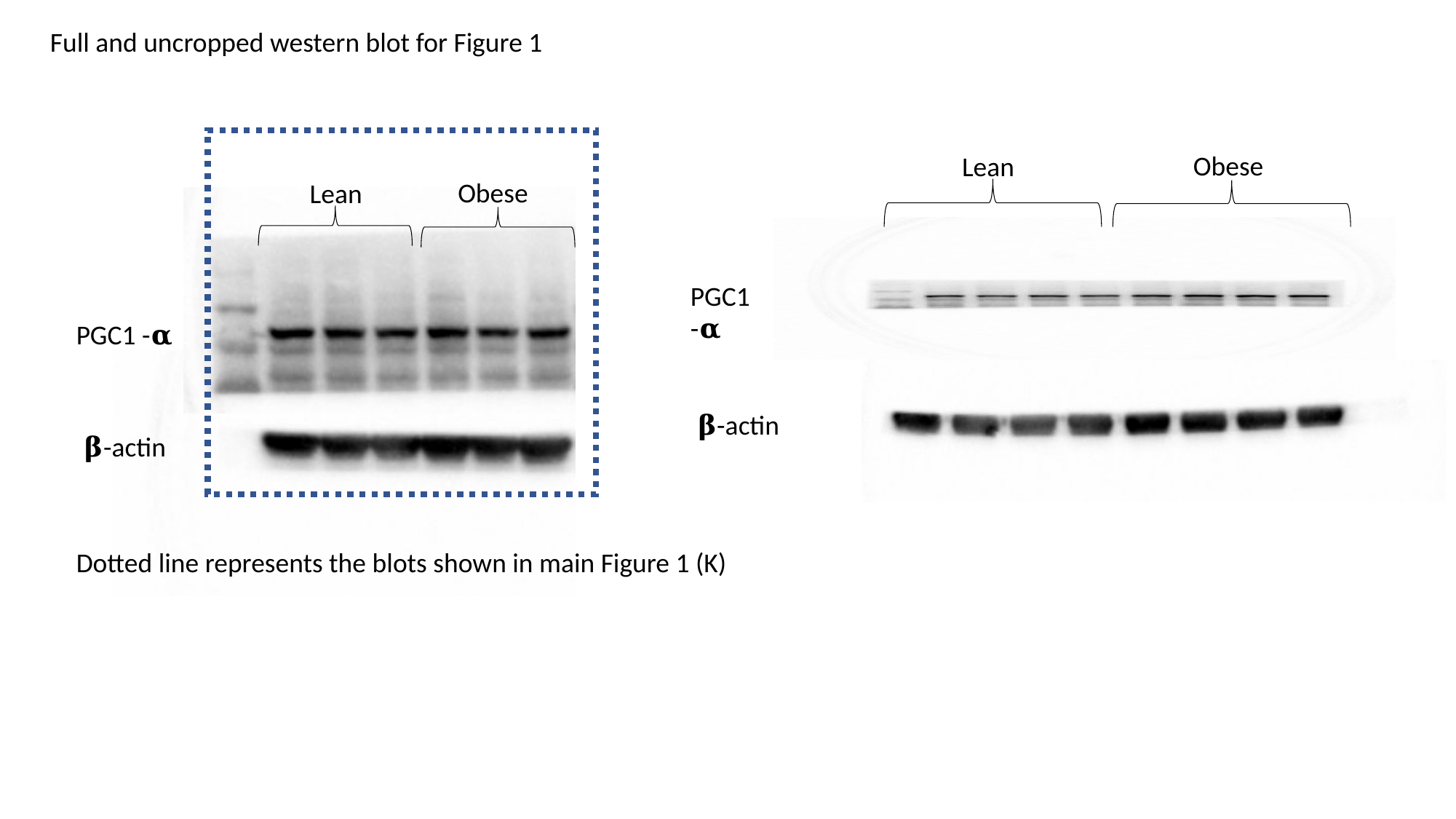

Full and uncropped western blot for Figure 1
Obese
Lean
PGC1 -𝛂
𝛃-actin
Dotted line represents the blots shown in main Figure 1 (K)
Obese
Lean
PGC1 -𝛂
𝛃-actin

## Slide 3
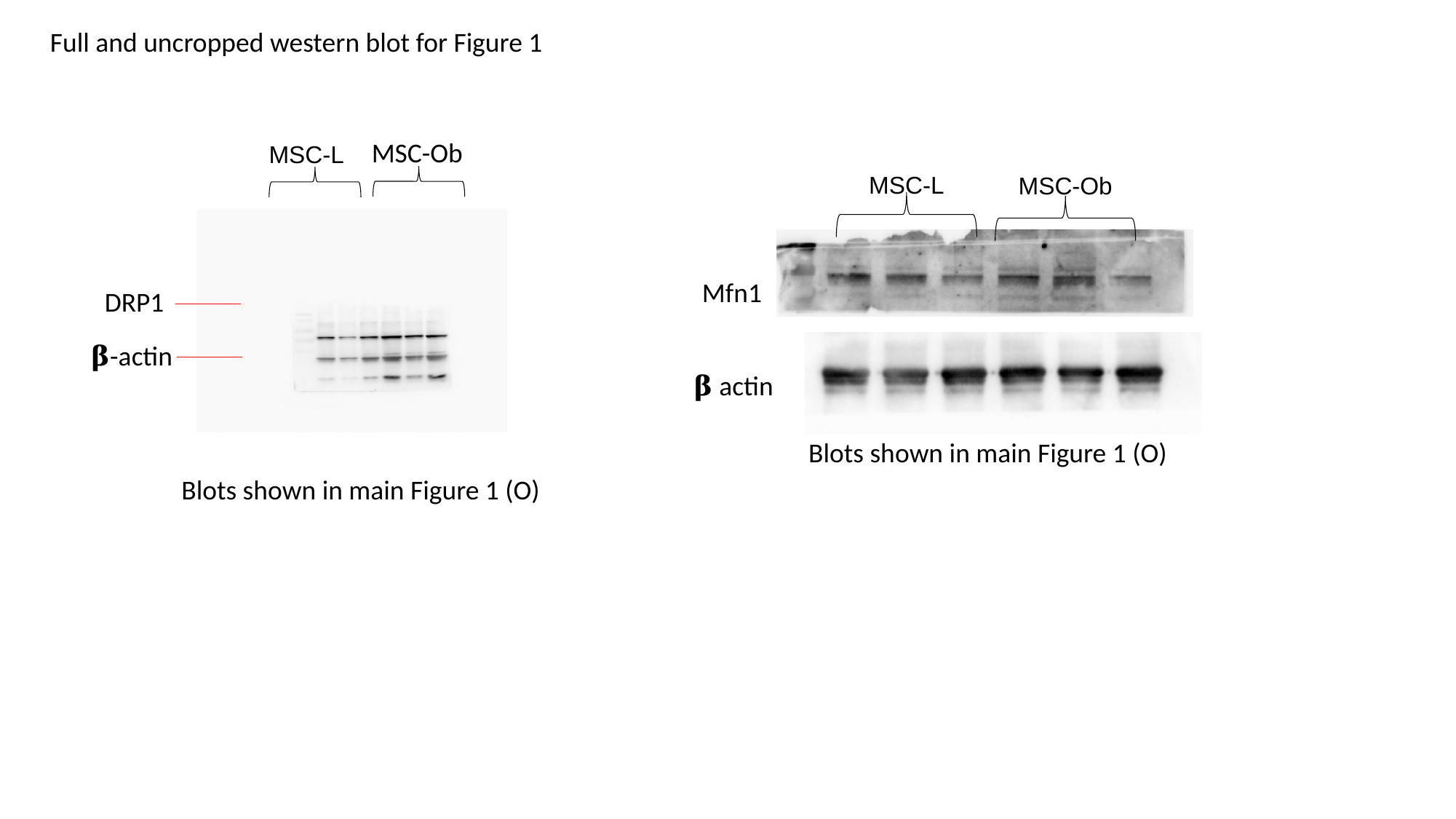

Full and uncropped western blot for Figure 1
MSC-Ob
MSC-L
DRP1
𝛃-actin
Blots shown in main Figure 1 (O)
MSC-L
MSC-Ob
Mfn1
𝛃 actin
Blots shown in main Figure 1 (O)

## Slide 4
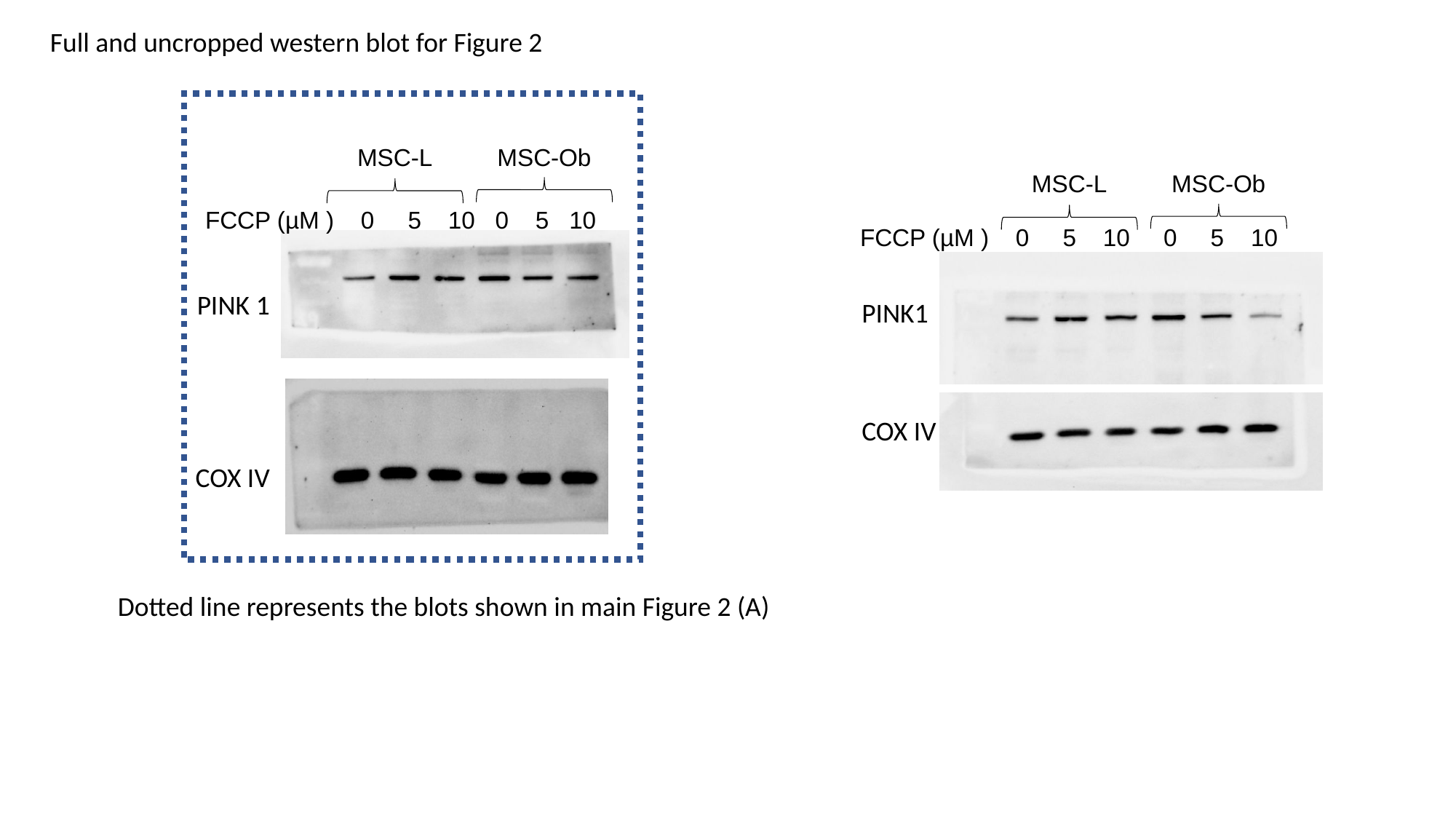

Full and uncropped western blot for Figure 2
MSC-L
MSC-Ob
FCCP (µM ) 0 5 10 0 5 10
PINK 1
COX IV
MSC-L
MSC-Ob
FCCP (µM ) 0 5 10 0 5 10
PINK1
COX IV
Dotted line represents the blots shown in main Figure 2 (A)

## Slide 5
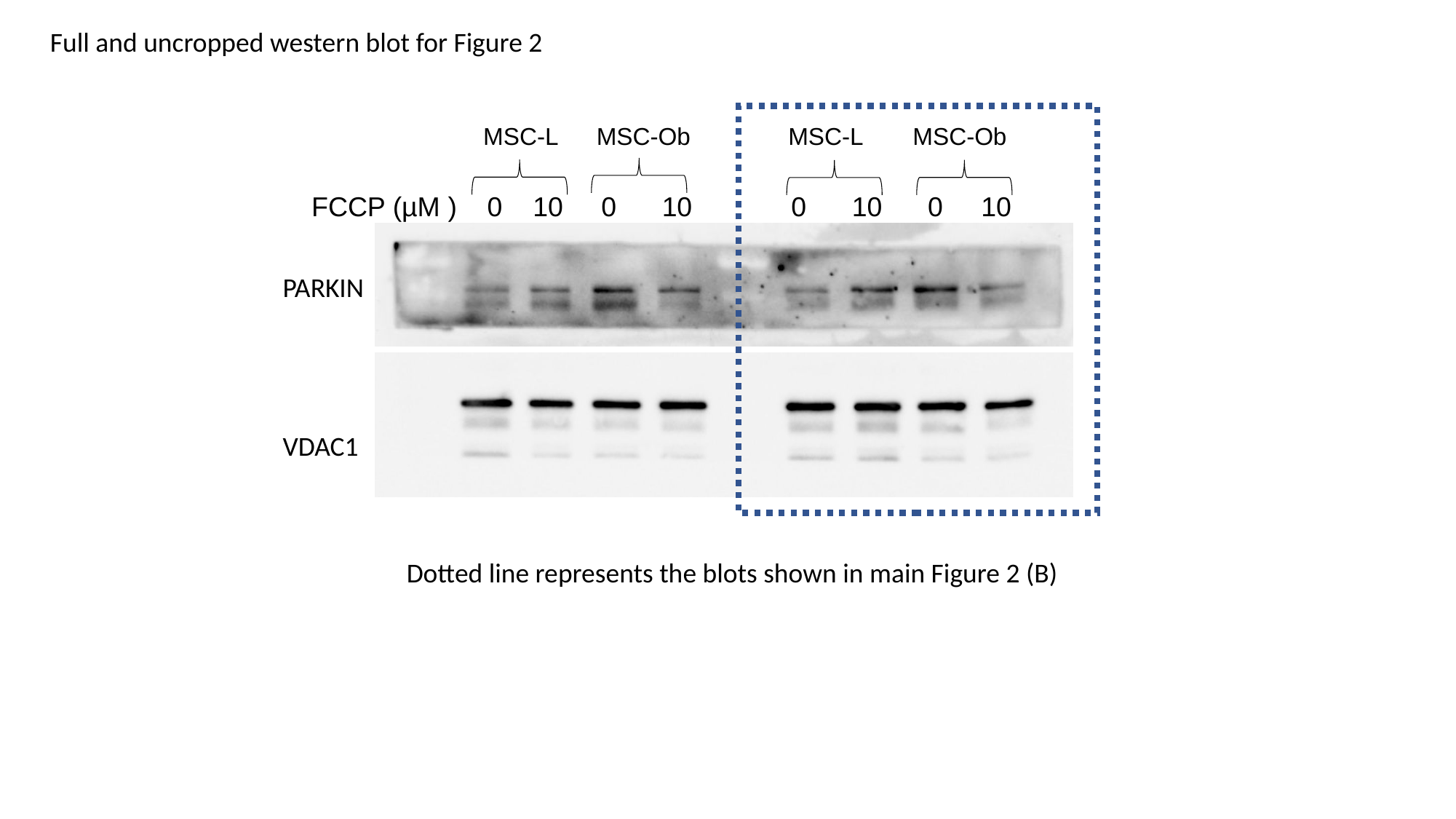

Full and uncropped western blot for Figure 2
MSC-L
MSC-Ob
MSC-L
MSC-Ob
FCCP (µM ) 0 10 0 10 0 10 0 10
PARKIN
VDAC1
Dotted line represents the blots shown in main Figure 2 (B)

## Slide 6
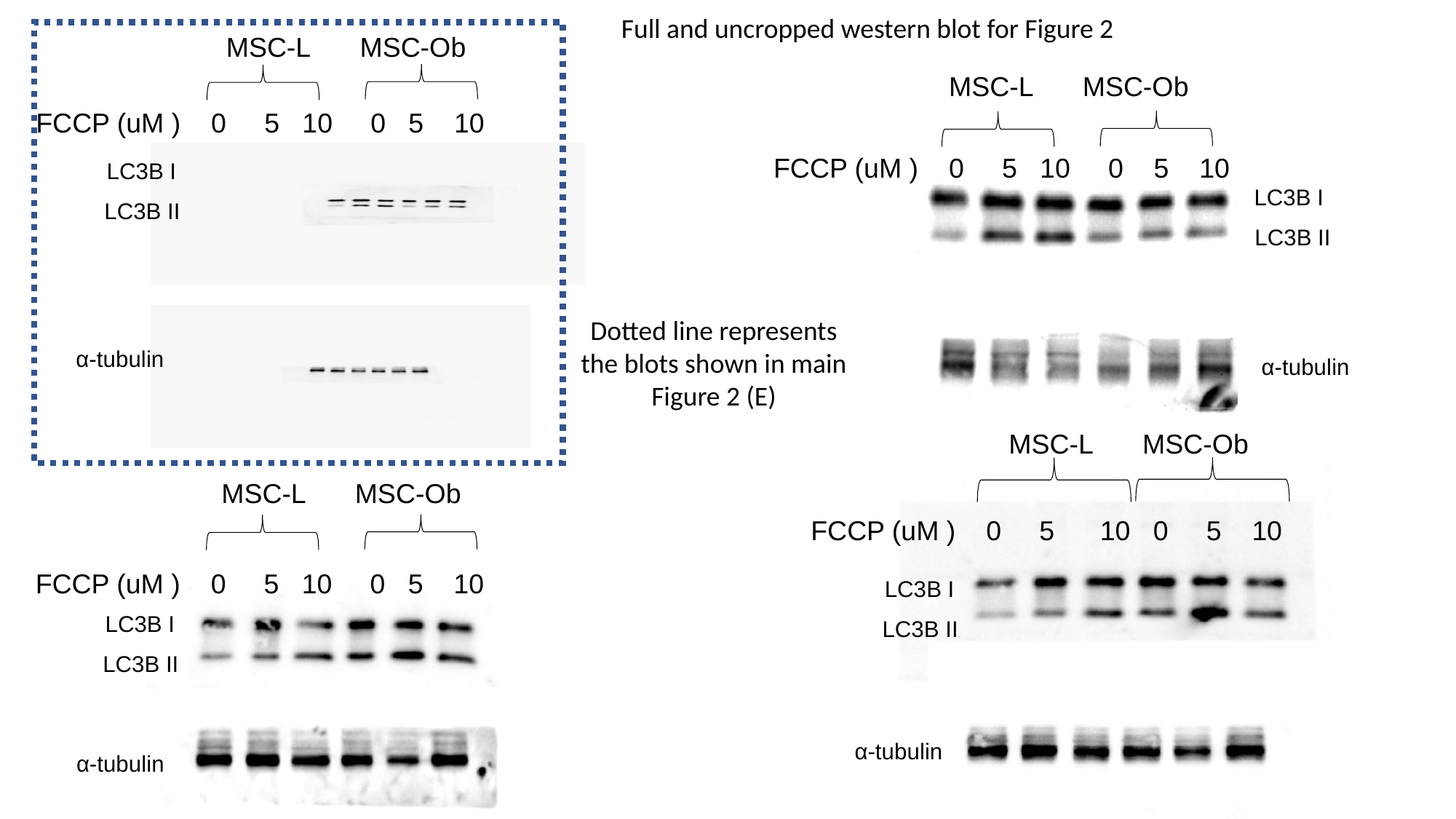

Full and uncropped western blot for Figure 2
MSC-L
MSC-Ob
MSC-L
MSC-Ob
FCCP (uM ) 0 5 10 0 5 10
FCCP (uM ) 0 5 10 0 5 10
LC3B I
LC3B I
LC3B II
LC3B II
Dotted line represents the blots shown in main Figure 2 (E)
α-tubulin
α-tubulin
MSC-L
MSC-Ob
MSC-L
MSC-Ob
FCCP (uM ) 0 5 10 0 5 10
FCCP (uM ) 0 5 10 0 5 10
LC3B I
LC3B I
LC3B II
LC3B II
α-tubulin
α-tubulin

## Slide 7
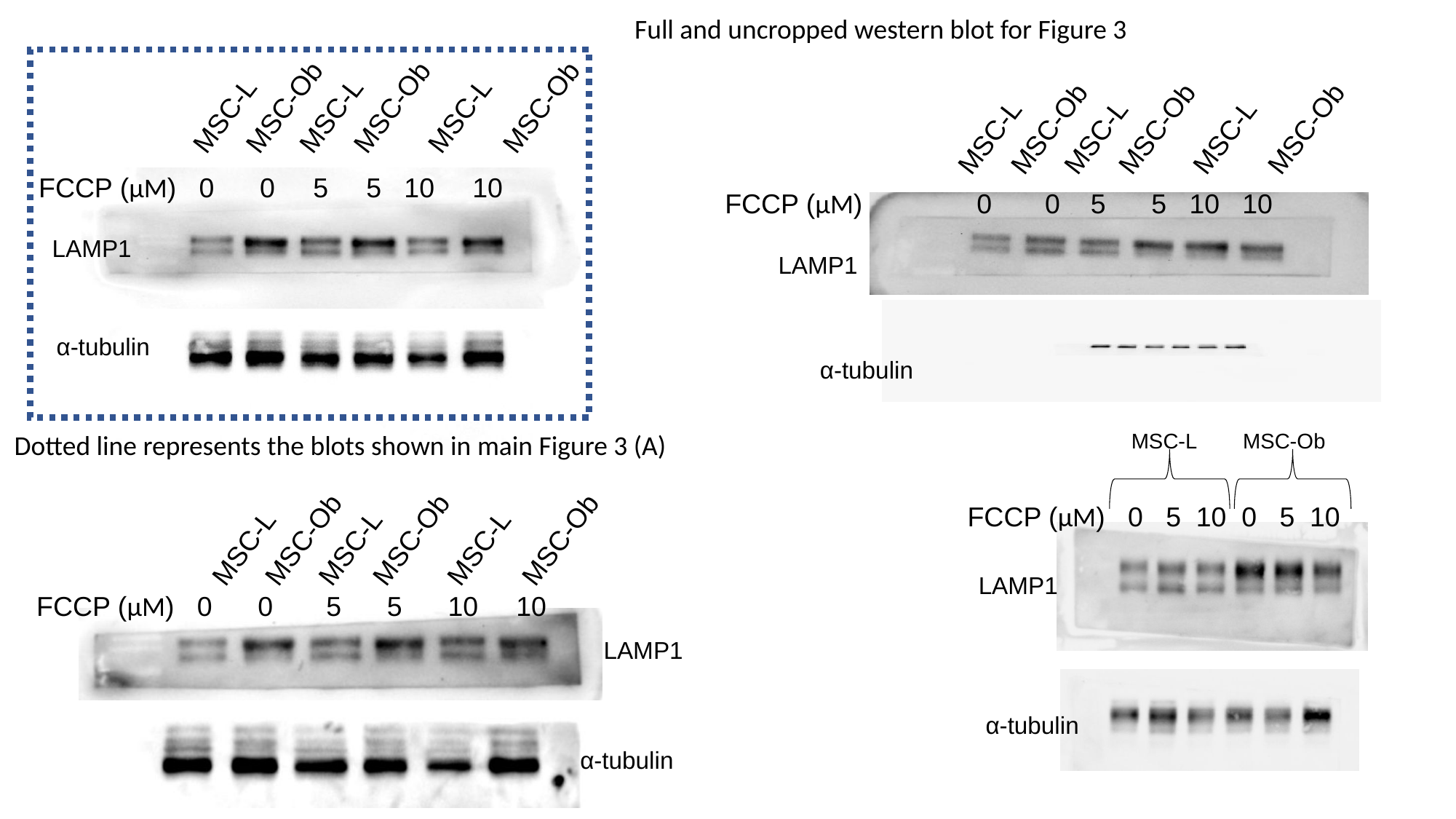

Full and uncropped western blot for Figure 3
MSC-Ob
MSC-Ob
MSC-Ob
MSC-L
MSC-L
MSC-L
LAMP1
α-tubulin
FCCP (μM) 0 0 5 5 10 10
MSC-Ob
MSC-Ob
MSC-Ob
MSC-L
MSC-L
MSC-L
FCCP (μM) 0 0 5 5 10 10
LAMP1
α-tubulin
MSC-L
MSC-Ob
FCCP (μM) 0 5 10 0 5 10
LAMP1
α-tubulin
Dotted line represents the blots shown in main Figure 3 (A)
MSC-Ob
MSC-Ob
MSC-Ob
MSC-L
MSC-L
MSC-L
FCCP (μM) 0 0 5 5 10 10
LAMP1
α-tubulin

## Slide 8
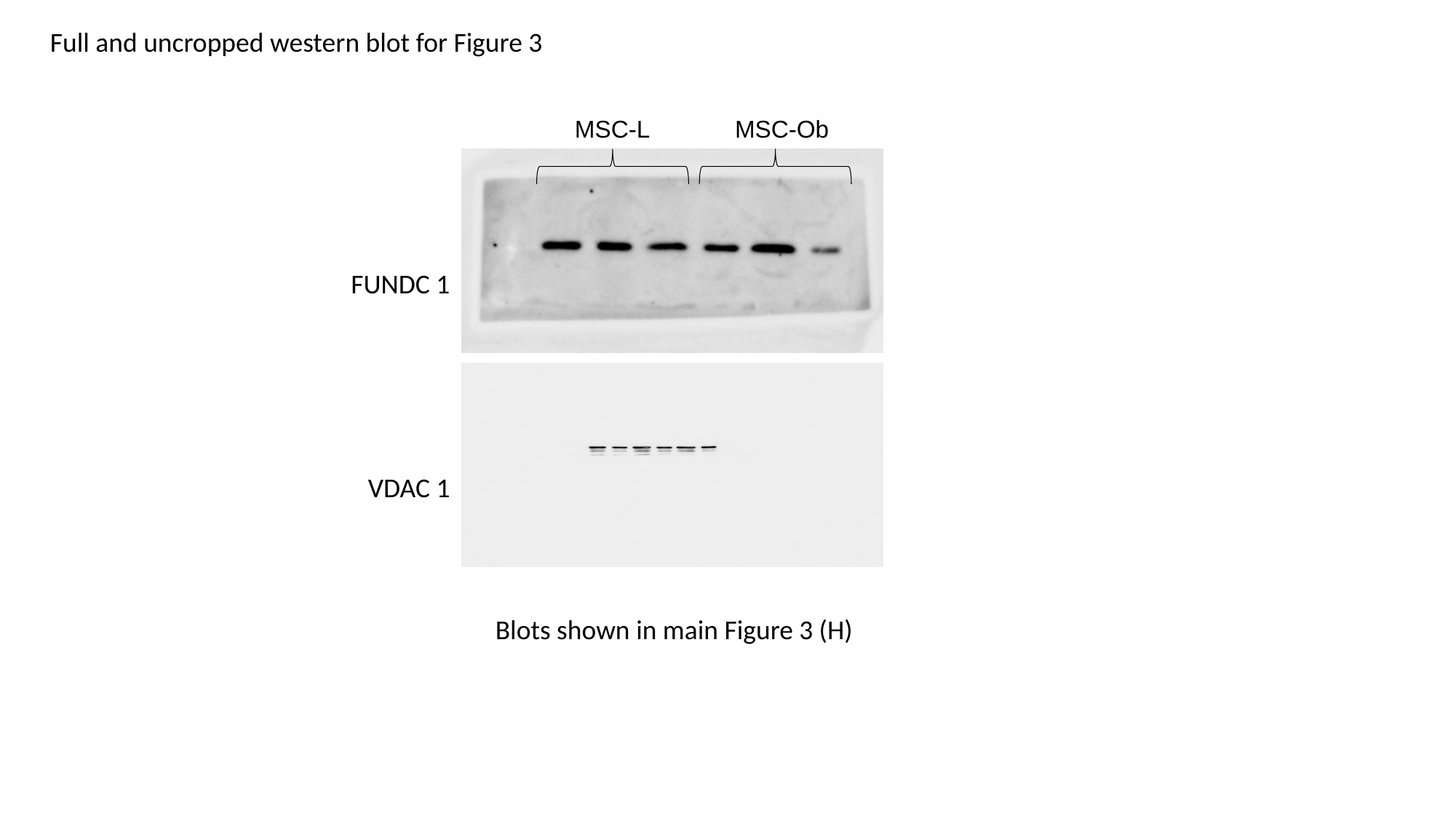

Full and uncropped western blot for Figure 3
MSC-Ob
MSC-L
FUNDC 1
VDAC 1
Blots shown in main Figure 3 (H)

## Slide 9
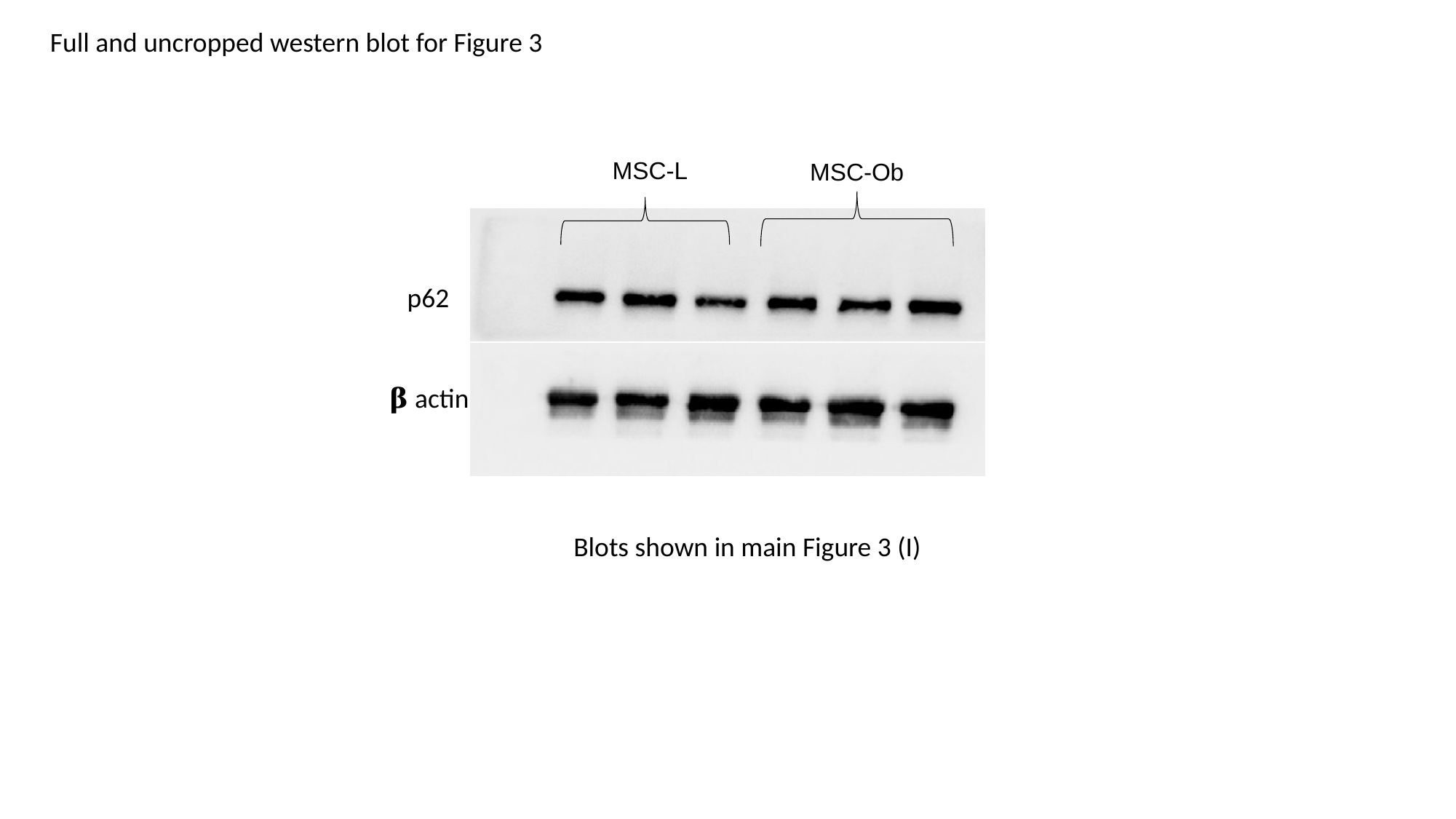

Full and uncropped western blot for Figure 3
MSC-L
MSC-Ob
p62
𝛃 actin
Blots shown in main Figure 3 (I)

## Slide 10
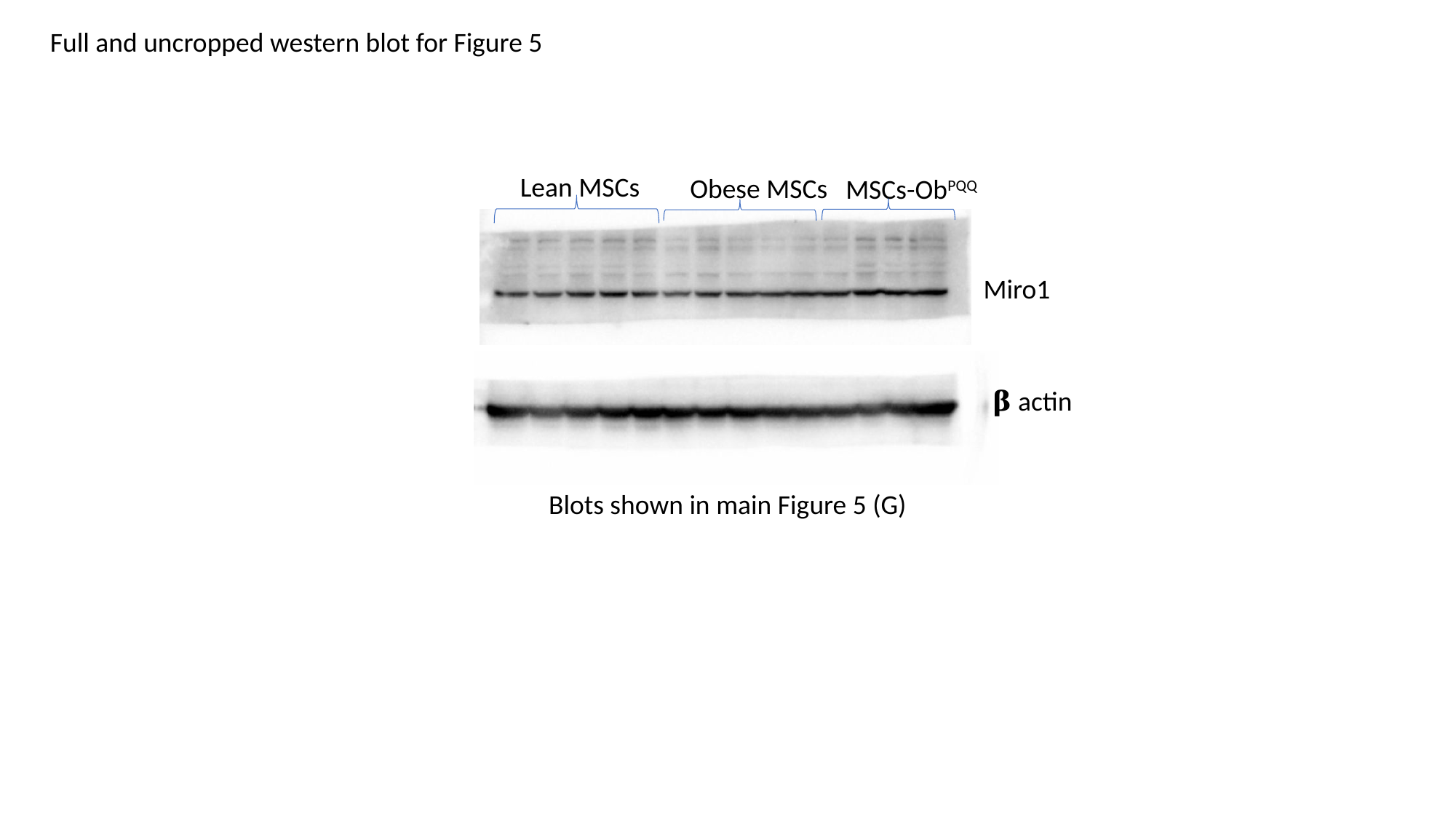

Full and uncropped western blot for Figure 5
Lean MSCs
Obese MSCs
MSCs-ObPQQ
Miro1
𝛃 actin
Blots shown in main Figure 5 (G)

## Slide 11
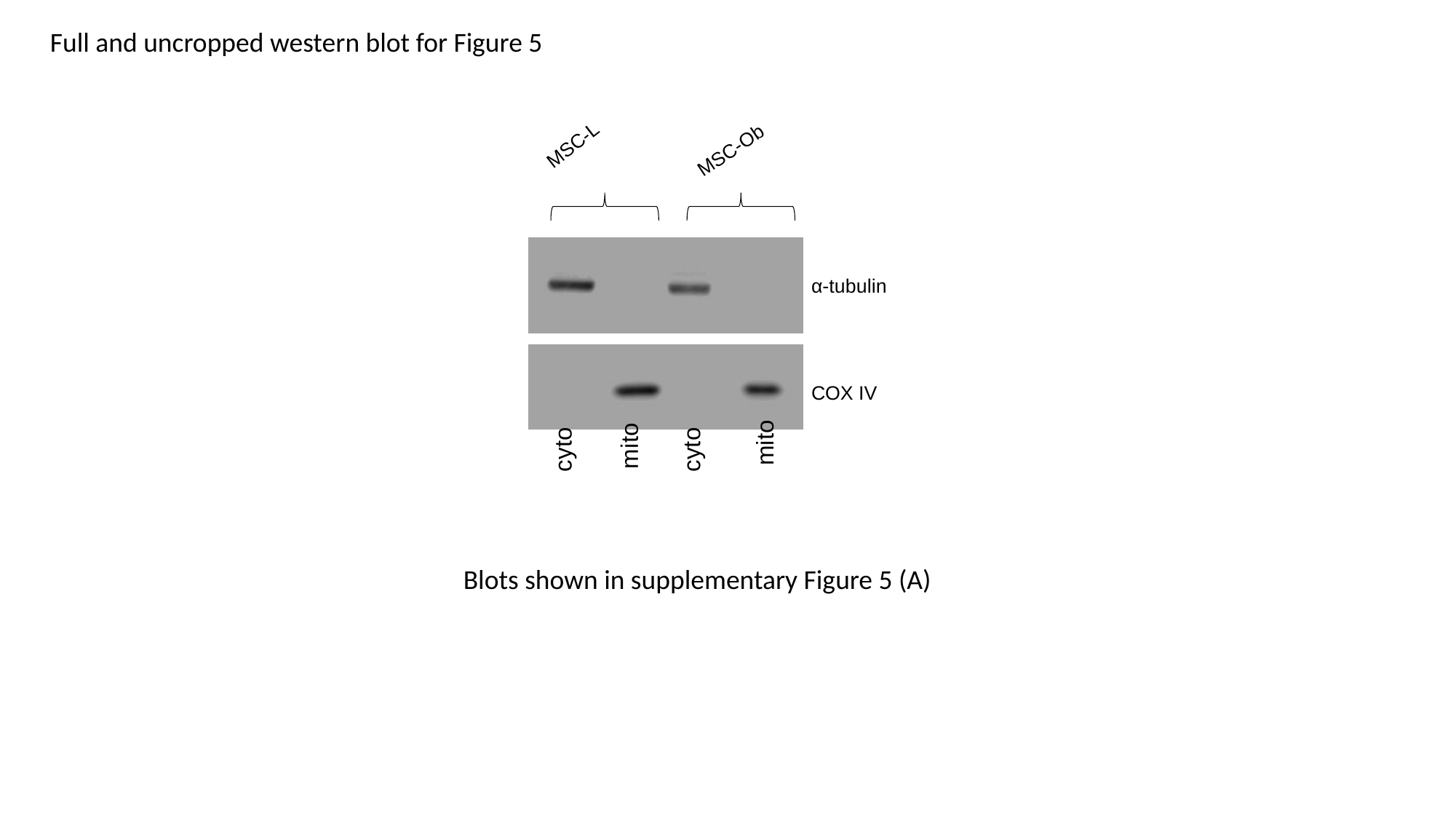

Full and uncropped western blot for Figure 5
MSC-L
MSC-Ob
α-tubulin
COX IV
mito
mito
cyto
cyto
Blots shown in supplementary Figure 5 (A)

## Slide 12
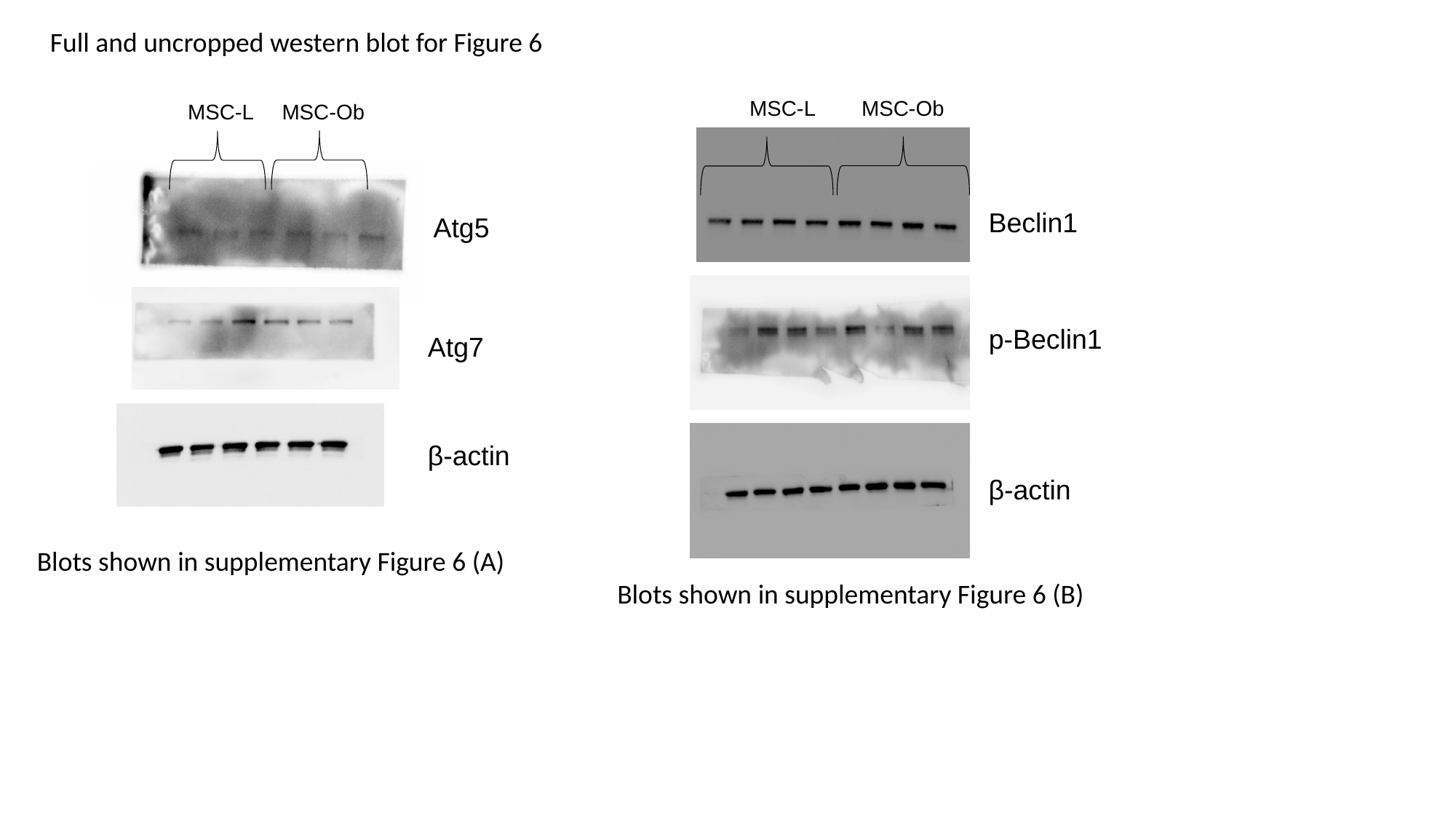

Full and uncropped western blot for Figure 6
MSC-L
MSC-Ob
Beclin1
p-Beclin1
β-actin
Blots shown in supplementary Figure 6 (B)
MSC-L
MSC-Ob
Atg5
Atg7
β-actin
Blots shown in supplementary Figure 6 (A)
